# Resistance exercise enhances long-term mTORC1 sensitivity to leucine

**DOI:** 10.1016/j.molmet.2022.101615

**Published:** 2022-10-14

**Authors:** Gommaar D'Hulst, Evi Masschelein, Katrien De Bock

**Affiliations:** Laboratory of Exercise and Health, Department of Health Sciences and Technology, Swiss Federal Institute of Technology (ETH) Zurich, Zürich, Switzerland

**Keywords:** Exercise, Leucine, Sensitivity, mTOR, ATF4

## Abstract

**Objective:**

Exercise enhances the sensitivity of mammalian target of rapamycin complex 1 (mTORC1) to amino acids, in particular leucine. How long this enhanced sensitivity lasts, and which mechanisms control enhanced leucine-mediated mTORC1 activation following exercise is currently unknown.

**Methods:**

C57BL/6J mice were exercised for one night in a resistance-braked running wheel after a 12-day acclimatization period. Mice were gavaged with a submaximal dose of l-leucine or saline acutely or 48 h after exercise cessation, following 3 h food withdrawal. Muscles were excised 30 min after leucine administration. To study the contribution of mTORC1, we repeated those experiments but blocked mTORC1 activation using rapamycin immediately before the overnight running bout and one hour before the first dose of leucine. mTORC1 signaling, muscle protein synthesis and amino acid sensing machinery were assessed using immunoblot and qPCR. Leucine uptake was measured using L-[^14^C(U)]-leucine tracer labeling.

**Results:**

When compared to sedentary conditions, leucine supplementation more potently activated mTORC1 and protein synthesis in acutely exercised muscle. This effect was observed in *m. soleus* but not in *m. tibialis anterio*r nor *m. plantaris*. The synergistic effect in *m. soleus* was long-lasting as key downstream markers of mTORC1 as well as protein synthesis remained higher when leucine was administered 48 h after exercise. We found that exercise enhanced the expression of amino acid transporters and promoted uptake of leucine into the muscle, leading to higher free intramuscular leucine levels. This coincided with increased expression of activating transcription factor 4 (ATF4), a main transcriptional regulator of amino acid uptake and metabolism, and downstream activation of amino acid genes as well as leucyl-tRNA synthetase (LARS), a putative leucine sensor. Finally, blocking mTORC1 using rapamycin did not reduce expression and activation of ATF4, suggesting that the latter does not act downstream of mTORC1. Rather, we found a robust increase in eukaryotic initiation factor 2α (eIF2α) phosphorylation, suggesting that the integrated stress response pathway, rather than exercise-induced mTORC1 activation, drives long-term ATF4 expression in skeletal muscle after exercise.

**Conclusions:**

The enhanced sensitivity of mTORC1 to leucine is maintained at least 48 h after exercise. This shows that the anabolic window of opportunity for protein ingestion is not restricted to the first hours immediately following exercise. Increased mTORC1 sensitivity to leucine coincided with enhanced leucine influx into muscle and higher expression of genes involved in leucine sensing and amino acid metabolism. Also, exercise induced an increase in ATF4 protein expression. Altogether, these data suggest that muscular contractions switch on a coordinated program to enhance amino acid uptake as well as intramuscular sensing of key amino acids involved in mTORC1 activation and the stimulation of muscle protein synthesis.

## Highlights

• Enhanced mTORC1 sensitivity to leucine persists up to 48 h after exercise.
• Increased muscle sensitivity to leucine is only observed in muscles which are recruited during exercise.
• Exercise drives a long-term increase in the expression of genes involved in amino acid transport, leading to improved leucine uptake.
• Acute exercise led to sustained expression of activated transcription factor 4 (ATF4), a main regulator of amino acid transporter/metabolism genes.
• mTORC1 suppression using rapamycin does not inhibit ATF4, suggesting that ATF4 is activated via stress responsive pathways.

## Graphical abstract

**Figure:**
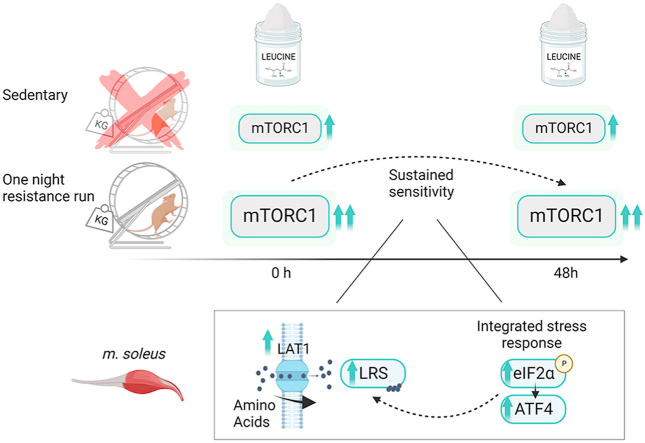

## 1 Introduction

Resistance exercise and dietary protein intake are important strategies for the retention of muscle mass across life span. A single bout of resistance training enhances both the rate of protein synthesis (MPS) and protein breakdown in skeletal muscle [1,2], but only when adequately high-quality protein is supplemented, protein balance shifts positive and optimal muscle remodeling can be expected [3,4]. Numerous studies have shown a synergistic effect of high-load muscular contractions and amino acid supplementation on protein synthesis [[5], [6], [7], [8]]. In those studies, proteins were always supplemented upon cessation or shortly after the exercise bout, leading to the postulation of an ‘anabolic window of opportunity’, a limited time frame after exercise during which synergistic exercise and amino acid effects on protein synthesis are achieved [9,10]. Intriguingly, more recent data indicate that enhanced sensitivity to amino acids persists multiple hours (12–24 hrs) after exercise cessation than initially proposed [11,12], but the mechanisms underlying prolonged amino acid sensitivity remain elusive.

In skeletal muscle, the main anabolic component of protein is leucine. Leucine triggers muscle protein synthesis independently from hyperaminoacidaemia [13] and the addition of leucine to a low protein beverage is as effective as a high-protein whey beverage at stimulating myofibrillar protein synthesis in healthy men [14]. How muscle fibers ‘sense’ leucine is complex, but emerging evidence indicates that fluctuations in intracellular leucine signal towards different components of the mammalian target of rapamycin complex 1 (mTORC1) [15,16]. mTORC1 drives the translation of mRNA species that encode for translation initiation factors, ribosomal proteins and mitochondrial proteins and contributes to muscle remodeling after protein ingestion or exercise [[17], [18], [19], [20]]. Currently, two main leucine sensors have been identified: leucyl tRNA synthetase (LARS) and stress response protein 2 (Sestrin2). LARS acts as the GAP of RagD, making it convert from the GTP- bound inactive form (RagDGTP) to the GDP-bound activated state (RagDGDP), to then trigger the activation of mTORC1 [16,21]. Sestrin2 has been defined as a putative leucine sensor in HEK-293T cells [22,23] via the inhibition of the GATOR2 subcomplex. However, the role of both leucine sensors in skeletal muscle has been questioned, as the dissociation constant (Kd) for LARS and sestrin2 for leucine is ∼10 fold lower than leucine concentrations in plasma from fasted humans [24], resulting in a complete saturation of both sensors under *in vivo* conditions. Yet, recent reports showed that acute exercise increased the expression of different Sestrin isoforms [25,26], as well as *Lars* mRNA transcripts in muscle [27]. Moreover, removing sestrin1/2 reduces the ability to inactivate mTORC1 in response to dietary leucine withdrawal [28]. This raises the intriguing question whether muscular contractions enhance the capacity to sense fluctuations in leucine by upregulating the expression of putative leucine sensors.

Acute exercise increases amino acid uptake during [29] and up to 3 h after exercise [2]. Selective amino acid transporters, such as members of the solute linked carrier (SLC) family, were upregulated up to 24 h after resistance training in human skeletal muscle [30]. Although the latter study did not assess amino acid uptake directly, these data point towards enhanced capacity to transport amino acids into the muscle after exercise cessation [30]. Nevertheless, it is currently unknown how long the potential beneficial effect of exercise on amino acid influx lasts and whether this may lead to an enhanced stimulation of mTORC1 and MPS when amino acids are ingested hours or even days after exercise. Thus, to fill this gap in knowledge, we sought to explore the mechanisms of increased leucine sensitivity after exercise towards mTORC1 using our previously described acute voluntary resistance mouse model [31] in combination with leucine feeding *in vivo* [32].

## 2 Methods

### 2.1 Animals

All animal procedures were approved by the Veterinary office of the Canton of Zürich (license nr. ZH137/2020). During the intervention, C57BL/6J mice were individually housed (22 °C, 12 h light/dark cycle, dark phase starting at 7 pm) in open cages equipped with a running wheel device (TSE Systems). Chow (18% protein, 4.5% fat, 66.7% carbohydrates, 4.5% fiber, and 6.3% ashes, Provimi Kliba SA) and water was provided ad libitum. Health status of all mouse lines was regularly monitored according to FELASA guidelines.

### 2.2 Experimental setup

**Figure 1:**
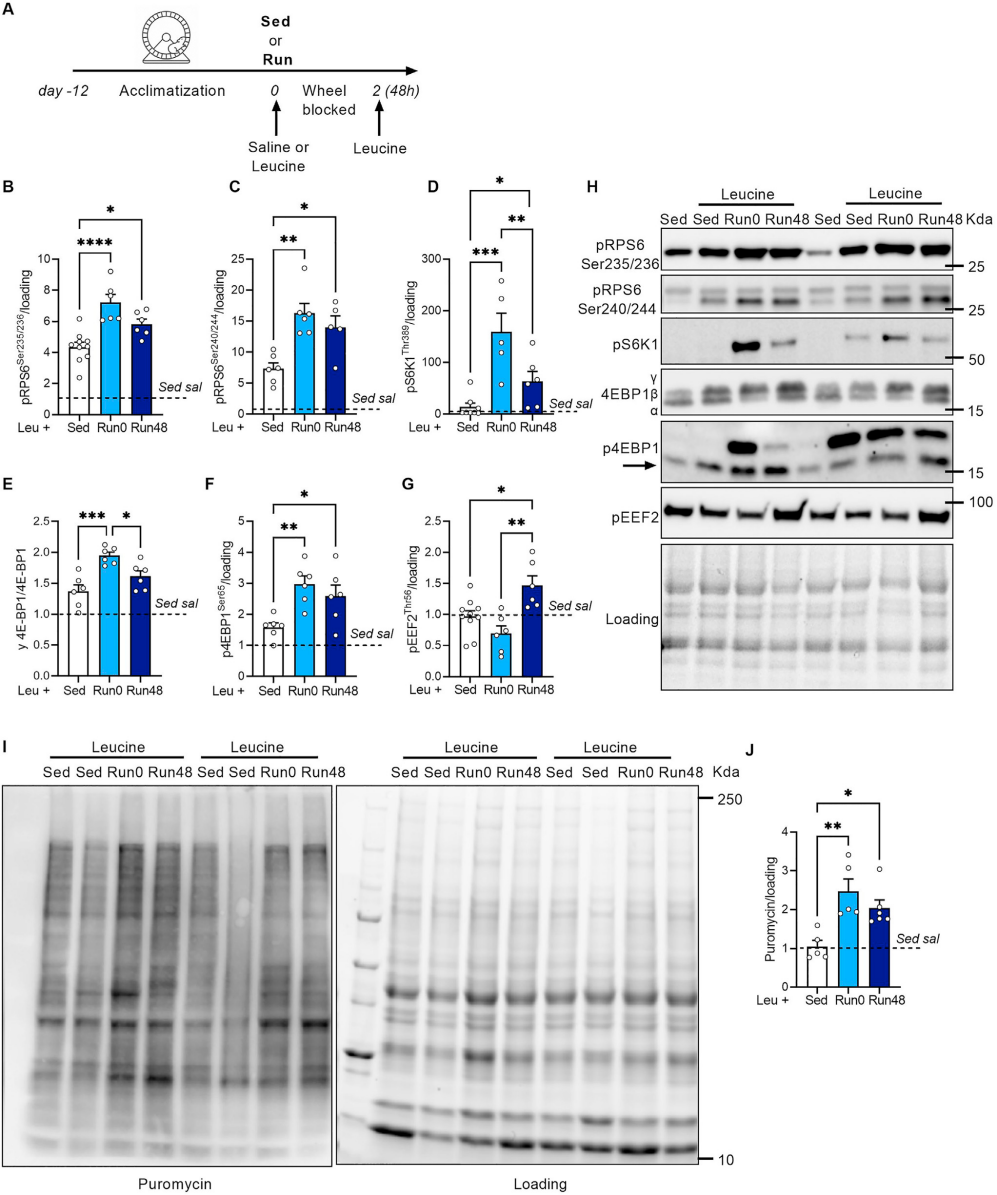
**Long-term activation of mTORC1 with exercise and leucine in SOL.** (A) Experimental set-up. Quantification (B–G) and representative images (H) of pRPS6^Ser235/236^ (B), pRPS6^Ser240/244^ (C), pS6K1^Thr389^ (D), γ-isoform of 4E-BP1 (E), 4E-BP1^Ser65^ (F), and pEEF2^Thr56^ (G). (I–J) Representative immunoblot (I) and quantification (J) of puromycin incorporation. Bars represent mean, circles represent individual values, error bars represent standard error of mean (SEM). All data is shown as fold change to Sed sal (dashed horizontal line). Panel B–G and J, (Sed sal n = 8), (Sed leu n = 10) (Run0 n = 6) (Run48 n = 6). One-Way ANOVA with Tukey’s multiple comparisons test (panel B–G and J). ∗ p < 0.05; ∗∗ p < 0.01, ∗∗∗p < 0.001, ∗∗∗∗p < 0.001.

An overview of the experimental set-up is provided in Figure 1A. All mice (12-14-week-old female C57BL/6J) underwent a 12-day acclimatization period to get accustomed to the wheel as described before [31]. Briefly, mice got access to the running wheel with 0% braking resistance during the first night, 20% braking resistance during the fourth night and 40% braking resistance during the seventh night. During the second, third, fifth, and sixth night, the running wheels were blocked. After the seventh night, mice remained in the (locked) resistance wheel cages for four additional nights. All mice underwent these familiarization sessions. Next, mice were randomly assigned to resistance exercise groups (Run) or sedentary groups (Sed). During the actual experimental run (the twelfth night), only the mice in the resistance exercise group (Run) had access to an unlocked wheel set at 60% resistance. In the morning after the experimental running bout, mice were food deprived for 3 h after which a dose of l-leucine (0.4 g kg^−1^; Leu) or saline (0.9% NaCl in H_2_O; Sal) was administered via oral gavage to the Sed and Run (Run0). Another group received an oral dose of l-Leucine 48 h after the last running bout (Run48). Mice in Sed group (both saline as well as leucine administration) were randomly assigned to experimental days 0 or 48 (combined with Run0 and Run48 experiments). Because we, as expected (data not shown), did not observe differences between sampling days, those samples were pooled. In Run48 groups, the wheel remained blocked in the 48 h time-window, but mice had ad libitum access to the chow diet (Figure 1A). Exactly 30 min after leucine or saline administration, mice were anaesthetized using Ketamine/Xylazine (115 μg g^−1^ and 13 μg g^−1^ bodyweight respectively) via intraperitoneal injection. Subsequently, the m. gastrocnemius (GAS), m. tibialis anterior (TA), m. soleus (SOL), were dissected and snap frozen for further analysis. After sample collection, animals were euthanized.

To investigate the effect of rapamycin, mice underwent a similar acute voluntary resistance running bout after a 12-day acclimatization period but were injected with DMSO (control) or 1.5 mg kg^−1^ rapamycin (Sigma–Aldrich, 553,210) dissolved in DMSO 2 h after running cessation and food withdrawal. Subsequently, leucine (0.4 g kg^−1^) was gavaged (1 h or 48 h after DMSO/rapamycin injection, each time 3 h after food withdrawal) and hindlimb muscles were dissected and snap frozen in liquid nitrogen for further analysis. In a follow-up set of experiments, the same dose of rapamycin was given immediately before the overnight run (6 PM, start of dark phase), as well as 2 h after running.

Food intake was assessed in a separate experiment by weighing food before the overnight run and 48 h after exercise. Mice were single caged throughout the experiment.

*In vivo* rate of protein synthesis was measured using the SUnSET method [33]. The mice were intraperitoneally injected with 0.04 μmol puromycin/g body weight (Sigma–Aldrich, P8833), exactly 30 min after injection, hindlimb muscles were dissected and snap frozen in liquid nitrogen for further analysis.

### 2.3 Skeletal muscle leucine uptake

An identical experimental set-up as depicted in Figure 1A was used. Mice were gavaged with bolus of 0.4 g kg^−1^ l-Leucine or saline spiked with 1.5 μCi l-[14 C(U)]-Leucine (PerkinElmer, NEC279E050UC). Blood samples were obtained from the tail vein before and 5, 15, and 30 min after administration of the solution (Figure 3G). Once the final blood sample was taken, mice were sacrificed and SOL, GAS and TA were quickly dissected and processed for further analysis. Muscle proteins were extracted by digesting muscle samples in lysis buffer (50 mM Tris–HCl pH 7.0, 270 mM sucrose, 5 mM EGTA, 1 mM EDTA, 1 mM sodium orthovanadate, 50 mM glycerophosphate, 5 mM sodium pyrophosphate, 50 mM sodium fluoride, 1 mM DTT, 0.1% Triton-X 100 and 10% protease inhibitor) using an OMNI-THq Tissue homogenizer (OMNI International) for 20 s until a consistent homogenate was formed. Samples were centrifuged for 10 min at 10,000 g and supernatant was used to assess radioactivity. Protein concentration was determined using the DC assay protein method (Biorad Laboratories) to equalize the amount of protein. Radioactivity was determined in 50 μl of this mixture in duplicate or in 5 μl serum by liquid scintillation counting (Beckman LS 6500; Beckman Coulter). The rate of l-leucine uptake (Kin) was calculated by the equation Kin = total dpm muscle protein/AUC 0–30 min.

**Figure 2:**
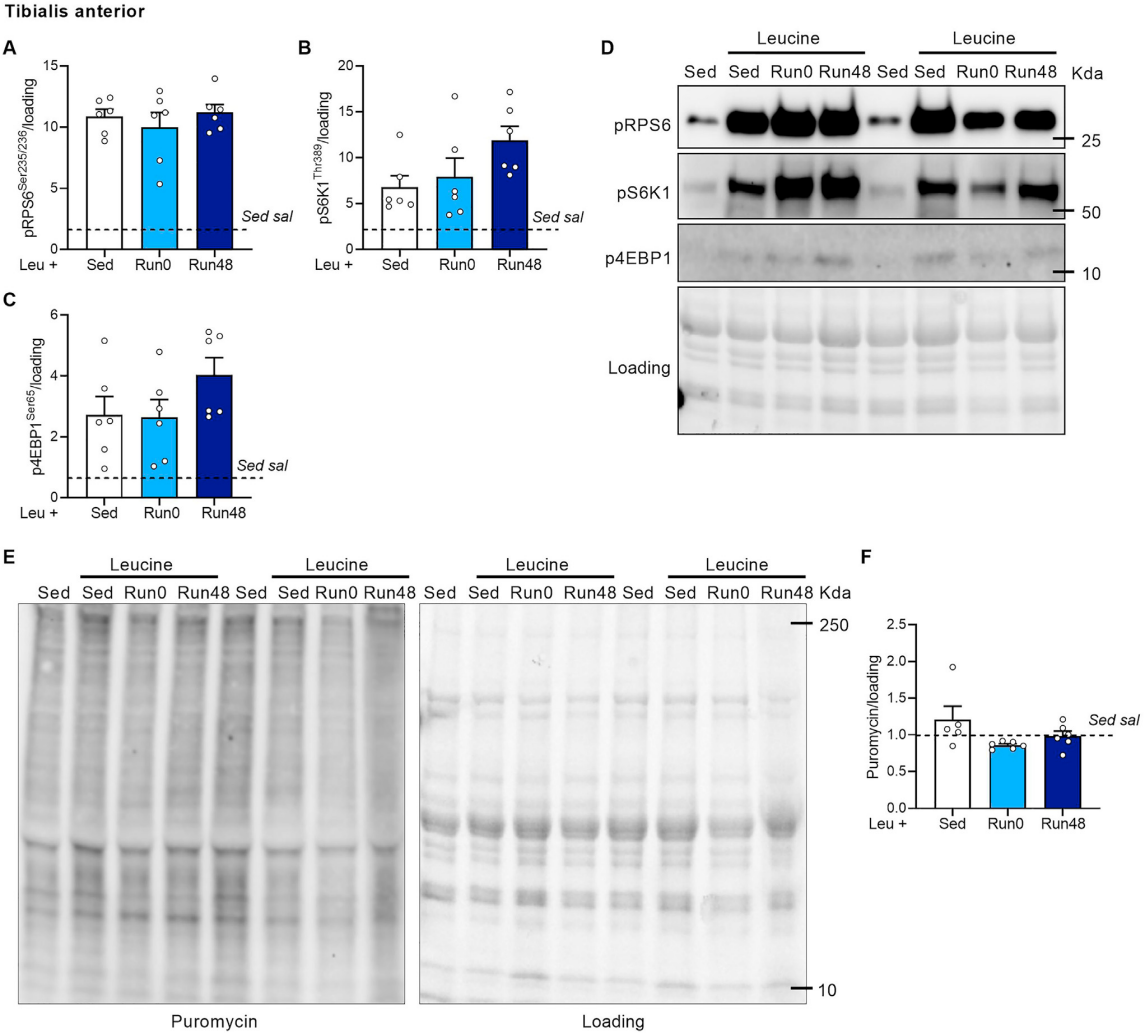
**No long-term activation of mTORC1 with exercise and leucine in TA.** Quantification (A–C) and representative immunoblots (D) of pRPS6^Ser235/236^ (A), pS6K1^Thr389^ (B), and 4E-BP1^Ser65^ (C). (E–F) Representative immunoblot (E) and quantification of puromycin incorporation. Bars represent mean, circles represent individual values, error bars represent standard error of mean (SEM). All data is shown as fold change to Sed sal (dashed horizontal line). Panel A–C and F, (Sed sal n = 6), (Sed leu n = 6) (Run0 n = 6) (Run48 n = 6). One-Way ANOVA with Tukey's multiple comparisons test (panel A–C and F). ∗ p < 0.05; ∗∗ p < 0.01, ∗∗∗p < 0.001.

**Figure 3:**
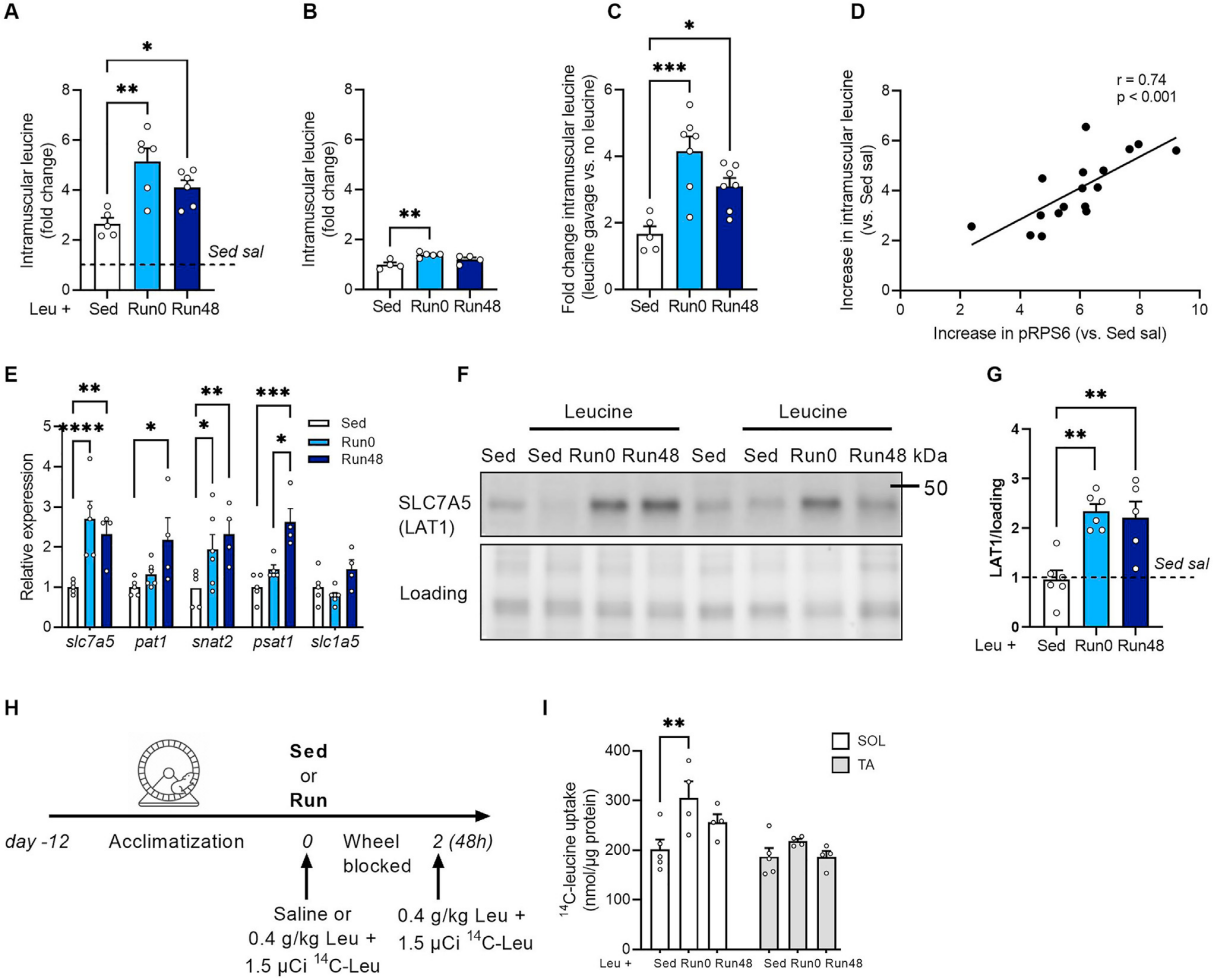
**Acute exercise drives long-term increase in leucine uptake.** (A) Intramuscular free leucine in SOL after exercise combined with leucine administration. (B) Intramuscular free leucine in SOL after exercise without leucine. (C) Fold change intramuscular free leucine in conditions where leucine was administered vs. no leucine (data derived from panel A and B). (D) Correlation between the increase in intramuscular free leucine (fold change versus Sed sal) and the increase in downstream mTORC1 signaling (pRPS6^Ser235/236^ versus Sed sal). (G) qPCR of amino acid transporter genes in SOL in sed, Run0 and Run48 conditions without leucine. Quantification and representative immunoblot for SLC7A5 (LAT1) in SOL (E–F). (H) Experimental set-up to assess ^14^C leucine uptake. (I) Leucine uptake in *m. soleus* (SOL) and *m. tibialis anterior* (TA) after exercise and leucine supplementation. Bars represent mean, circles represent individual values, error bars represent standard error of mean (SEM). All data is shown as fold change to Sed sal (dashed horizontal line, when applicable). Panel A, (Sed sal n = 6), (Sed leu n = 6) (Run0 n = 6) (Run48 n = 6). Panel B, (Sed leu n = 4) (Run0 n = 6) (Run48 n = 4). Panel E, (Sed leu n = 5) (Run0 n = 6) (Run48 n = 4). Panel G, (Sed leu n = 6) (Run0 n = 6) (Run48 n = 5). Panel I, (Sed leu n = 5) (Run0 n = 6) (Run48 n = 5). One-Way ANOVA with Tukey's multiple comparisons test (panel A–D, G and I). ∗ p < 0.05; ∗∗ p < 0.01, ∗∗∗p < 0.001.

### 2.4 Protein extraction and western blot

10 mg (SOL) and 25 mg (TA) of muscle sample was homogenized in ice cold lysis buffer (1:10, w/v) (50 mM Tris-HCl pH 7.0, 270 mM sucrose, 5 mM EGTA, 1 mM EDTA, 1 mM sodium orthovanadate, 50 mM glycerophosphate, 5 mM sodium pyrophosphate, 50 mM sodium fluoride, 1 mM DTT, 0.1% Triton-X 100 and 10% protease inhibitor) (20 μl per 1.8–2.5 μg of tissue sample) using an OMNI-THq Tissue homogenizer (OMNI International) for 20 s until a consistent homogenate was formed. Samples were centrifuged at 4 °C at 10,000 g for 10 min and the supernatant with proteins collected. Protein concentration was determined using the DC assay protein method (Biorad Laboratories) to equalize the amount of protein. An internal standard was added to each gel to compare samples between gels. Protein Transfer Samples were prepared 3:4 with laemmli buffer containing 10% 2-mercaptoethanol (Bio-rad laboratories) and heated at 95° for 5 min. Proteins were run on a 4–20% Mini-PROTEAN TGX Stain Free Pre-Cast Gel (Biorad Laboratories) for 45 min at 120–140 V. After electrophoresis, a picture of the gel was taken under UV-light to determine protein loading using strain-free technology. Proteins were transferred via semi-dry transfer onto a polyvinylidene fluoride membrane (Bio-rad, 170-4156) and subsequently blocked for 1 h at room temperature with 5% milk in TBS-Tween. Membranes were incubated overnight at 4 °C with primary antibodies listed in Table 1 (1:1000). The appropriate secondary antibodies (1:5000) for anti-rabbit and anti-mouse IgG HRP-linked antibodies (Cell signaling) were used for chemiluminescent detection of proteins. Membranes were scanned with a chemidoc imaging system (Bio-rad) and quantified using Image lab software (Bio-rad).

**Table 1:** Antibodies.

Antibody	Manufacturer	Reference
*p-S6K1^Thr389^*	*Cell signaling*	*9234*
*p-RPS6^Ser235/236^*	*Cell signaling*	*2211*
*Puromycin*	*Merk-millipore*	*MABE343*
*LARS*	*Cell signaling*	*13868*
*IleRS*	*Novus Biologicals*	*NBP1-87698*
*ATF4*	*Cell signaling*	*11815*
*p-4EBP1^Ser65^*	*Cell signaling*	*9451*
*p-eEF2^Thr56^*	*Cell signaling*	*2331*
*4EBP1*	*Cell signaling*	*9452*
*p-eIF2α^Ser51^*	*Cell signaling*	*3398*
*SLC7A5 (LAT1)*	*Protein Tech*	*13752-1-AP*

### 2.5 RT-qPCR

Muscle tissue (SOL, 10–15 mg) was homogenized with a tissue homogenizer (Omni THq) in 1000 μl ice-cold TRIzol (ThermoFisher Scientific, 15596018), and after addition of 200 μl chloroform, the homogenate was spun down for 15 min at 12,000*g*. The clear phase was mixed with 70% Ethanol and transferred into a mRNA extraction column (ThermoFisher Scientific, 12183018A). Subsequently, mRNA was extracted according to the manufacturer's instruction. Messenger RNA of cells was extracted using the same mRNA extraction kit. The purity and quantity of mRNA was assessed via a photospectrometer (Tecan, Spark). mRNA was reverse-transcribed using iScript cDNA synthesis kit (Bio-Rad, 170-8891). A SYBR Green-based master mix (ThermoFisher Scientific, A25778) was applied for real-time qPCR analysis. Primers used are listed in Table 2. To compensate for variations in mRNA input and efficiency of reverse-transcription, three housekeeping genes were used (GAPDH, β-Actin, 18S). All three housekeeping genes were assessed for between sample variation. β-Actin was used for all analyses because it showed to lowest SD (<0.5 CT) between samples and was not affected by treatment condition. The delta–delta CT method was used to normalize the data.

**Table 2:** Primer sequences.

Gene	Forward	Reverse
*atf4*	CAACCTATAAAGGCTTGCGG	CTGCTGGATTTCGTGAAGAG
*asns*	AAGCGAGCATCATGAAGTCC	AATACATGCCCACAGATGCC
*slc7a5*	CTCTTCCTCATTGCCGTGTC	CCGTCACAGAGAAGATAGCC
*phgdh*	GGTTACACAAGGAACATCTCTG	CTTAGCGTTCACCAAGTTCAC
*slc1a5*	TCTGCCTCTCATCTACTTCCT	CACACCATTCTTCTCCTCTAC
*snat2*	GGCATTCAATAGCACCGCAG	ACGGAACTCCGGATAGGGAA
*pat1*	CCGCTACCATGTCCACACAG	GGCCACGATACCAATCACCA
*gapdh*	ACCCAGAAGACTGTGGATGG	CACATTGGGGGTAGGAACAC
*18S*	AGTCCCTGCCCTTTGTACACA	CGATCCGAGGGCCTCACTA
*b-actin*	GTCCCAGACATCAGGGAGTAA	TCGGATACTTCAGCGTCAGGA

### 2.6 GC-MS determination of leucine levels

Extractions for subsequent mass-spectroscopy analysis were prepared and analyzed as previously described [34]. Flash frozen tissues were weighted and placed on lysing matrix vial containing 600 μl of extraction buffer. The tubes were homogenized three times for 20 s. Next, the lysing matrix tubes were centrifuged at 20,000×*g* for 10 min at 4 °C. 200 μl of supernatant was transferred to MS vials. Mass Spectrometry measurements were performed using Dionex UltiMate 3000 LC System (Thermo Scientific) coupled to a Q Exactive Orbitrap mass spectrometer (Thermo Scientific) operated in negative mode. 10 μl sample was injected onto a Poroshell 120 HILIC-Z PEEK Column (Agilent InfinityLab). A linear gradient was carried out starting with 90% solvent A (acetonitrile) and 10% solvent B (10 mM Na-acetate in mqH2O, pH 9.3). From 2 to 12 min the gradient changed to 60% B. The gradient was kept on 60% B for 3 min and followed by a decrease to 10% B. The chromatography was stopped at 25 min. The flow was kept constant at 0.25 ml/min. The columns temperature was kept constant at 25 °C. The mass spectrometer operated in full scan (range [70.0000–1050.0000]) and negative mode using a spray voltage of 2.8 kV, capillary temperature of 320 °C, sheath gas at 45, auxiliary gas at 10. AGC target was set at 3.0E+006 using a resolution of 70,000. Data collection was performed using the Xcalibur software (Thermo Scientific). The data analyses were performed by integrating the peak areas (El-Maven – Polly - Elucidata).

### 2.7 Statistical and data analyses

Results are presented as mean with standard error of the mean (SEM) bars and individual data points. If applicable, Sed mice which received a saline gavage (Sed sal) are represented in the figures as a dashed line at the relative value of 1.0. The values of all other groups are relative to Sed sal. Leucine-treated groups were subjected to a one-way analysis of variance (ANOVA) and when appropriate post hoc tests were performed using Tukey's post hoc test using Graphpad Prism 9.2 to compare between groups. Two-way analysis of variance (ANOVA) with appropriate Tukey's post hoc test were used to assess time-dependent effects of leucine. Significance was set at p < 0.05.

## 3 Results

### 3.1 Enhanced mTORC1 sensitivity to leucine persists up to 48 h after exercise

Acute exercise enhances the sensitivity of mTORC1 to amino acids, in particular BCAAs [35]. How long this enhanced sensitivity lasts is not known. We used a mouse model of resistance running to investigate whether leucine supplementation sustains the increase in mTORC1 activity acute and long-term after exercise (Figure 1A). In agreement with previous studies in humans [[36], [37], [38]], we found that exercise (Run0) combined with leucine supplementation had a synergistic effect on mTORC1 activation in *m. soleus* (SOL) as the combination increased pRPS6^Ser235/236^ and ^Ser240/244^ more when compared to leucine alone (Figure 1B,C,H). Other downstream mTORC1 targets pS6K1^Thr389^ and 4EBP1 showed similar patterns (Figure 1D–F,H). Interestingly, this synergistic effect was long-lasting as leucine administration 48 h after exercise (Run48) increased key downstream markers of mTORC1 activity more than leucine alone without exercise (Sed). Nonetheless, the sensitizing effect diminished slightly over time as mTORC1 signaling was lower in Run48 versus Run0 (Figure 1B–F,H). On the other hand, phosphorylation of eEF2 at Thr56, which inhibits its activity [39], only increased after leucine in Run48 (Figure 1G,H), again suggesting long-term effects of exercise on mTORC1. Importantly, Run0 robustly increased baseline mTORC1 activity, but this almost completely returned to baseline levels at Run48 (Fig. S1A–D). Indeed, we observed slightly higher pRSP6^Ser240/244^ at Run48 but this was not the case for pS6K1^Thr389^. Moreover, the increase in pS6K1^Thr389^ to leucine was higher at Run0 as well as Run48 (interaction effect, Figure 1B,D), further underscoring that the response to leucine was amplified in Run0/48. It is worthwhile mentioning that the interaction did not reach significance in pRSP6^Ser240/244^ despite consistent higher levels upon leucine supplementation in Run0/48 compared to Sed (Fig. S1C–D). Moreover, mice that were running had a slight increase in food intake (Fig. S1H; p = 0.07)

Finally, to assess whether enhanced mTORC1 activity resulted into higher protein synthesis, we measured protein synthesis using the SUnSET puromycin incorporation assay [40]. Leucine supplementation robustly increased puromycin incorporation in Run0 and Run48, while leucine in Sed did not enhance puromycin incorporation in SOL (Figure 1I,J). In fact, even though we observed a small decline in protein synthesis over time, this failed to reach significance, showing that the synergistic effects of exercise and leucine administration on mTORC1 activity and protein synthesis are robust and long-lasting.

### 3.2 No prolonged enhanced mTORC1 sensitivity after exercise in *m. plantaris* (PLT) nor *m. tibialis anterior* (TA)

Earlier work from our group has shown that resistance running only, without leucine supplementation, increases mTORC1 signaling in plantar flexors such as SOL and to a lesser extent in *m. plantaris* (PLT) [31], which has a higher proportion of fast glycolytic fibers. First, we observed a very strong activation of mTORC1 upon leucine administration in Sed conditions, suggesting that PLT is particularly sensitive to leucine (Fig. S1E–G). Moreover, in agreement with our previous work, we observed that exercise increased mTORC1 activity under baseline (Saline in Run0) conditions, confirming its recruitment during exercise (Fig. S1E–G). Interestingly, we did not observe a synergistic effect of leucine supplementation at RUN0 and RUN48 in PLT (Fig. S1E–G).

We also previously showed that resistance running does not increase mTORC1 signaling in dorsi flexors such as *m. tibialis anterior* (TA), which are recruited to a limited extent during resistance wheel running [31]. As a consequence, 8-weeks of voluntary resistance running only induced hypertrophy in the plantar flexors [41,42], while the dorsi flexors remained largely unaffected [31]. In accordance with these data, we found that exercise did not sensitize the TA to leucine at any time point, not acute, nor 48 h after exercise. Leucine increased pRPS6^Ser235/236^ to a similar extent in both the Run mice as well as the Sed mice (Figure 2A,D). Other mTORC1 downstream targets pS6K1^Thr389^ and p4EBP1^Ser65^ showed similar patterns (Figure 2B–D). Furthermore, puromycin incorporation was not affected by leucine administration, nor by exercise (Figure 2E,F).

Altogether, these data show that the sensitizing effect of exercise towards leucine is muscle specific and depends on the activation pattern of the muscle and potentially fiber type composition.

### 3.3 Acute exercise drives a long-term increase in leucine uptake

To explore the potential mechanisms underlying the exercise-induced increase in leucine sensitivity, we decided to assess intramuscular leucine levels, since those critically control mTORC1 activity [43]. Exercise acutely increases amino acid uptake in the muscle [2,44], but whether this effect is still present 48 h after exercise cessation, and how it is related to mTORC1 activation, is unknown. To address this, we first measured free leucine levels in SOL via GC-MS. Leucine supplementation to sedentary mice increased intramuscular free leucine concentrations by ∼2-fold compared to saline control. Feeding the same amount of leucine (0.4 g kg^−1^ bw) at Run0 and Run48 led to higher free leucine levels when compared to Sed (Figure 3A). This was not secondary to an increase in baseline leucine levels since those were only modestly elevated in Run0 mice and returned to Sed levels in Run48 (Figure 3B). Thus, the increase in muscle free leucine levels (Δ leucine) upon leucine supplementation was higher in Run0 when compared to Sed and remained elevated at Run48 (Figure 3C). Interestingly, the rise in intramuscular free leucine and the increase in mTORC1 downstream target (pRPS6) with leucine supplementation were highly correlated (Figure 3D), suggesting that changes in intracellular leucine are closely related to mTORC1 activation both under sedentary and exercise conditions.

To evaluate whether the increased intramuscular leucine levels upon leucine administration could be caused by enhanced leucine uptake, we first assessed the expression of *slc7a5*, a heterodimeric transporter complex responsible for uptake of large neutral amino acids in various tissues, including skeletal muscle [45]. Indeed, muscle-specific *slc7a5* knock-out mice show impaired leucine mediated mTORC1 activation [45]. We found a robust and prolonged increase of *slc7a5*/LAT1 acutely and 48 h after exercise, both on mRNA and protein level (Figure 3E–G). mRNA expression of several other amino acid transporters were also upregulated at Run0 and retained higher expression at Run48 (Figure 3G). This shows that acute resistance running leads to a sustained (at least 48 h) increase in amino acid transporter expression in skeletal muscle, potentially leading to increased leucine accumulation upon leucine supplementation. To further corroborate these findings, we assessed leucine uptake. To do so, we spiked the leucine bolus (0.4 g kg^−1^ bw) with a l-[^14^C(U)]-leucine tracer and supplemented this under identical experimental conditions as described above (Figure 3H). Since leucine and other branched chain amino acids are rapidly (within minutes) oxidized in muscle and undergo fast transamination [46], thereby preventing reliable assessment of uptake kinetics, we decided to measure the stable incorporation of exogenous (tracer labeled) leucine into proteins. Exercise (Run0) increased leucine uptake in muscle when compared to Sed (Figure 3I). Leucine uptake in Run48 remained elevated compared to sedentary control in SOL, even though it slightly decreased when compared to Run0 (Figure 3I). Remarkably, the increase in post-exercise leucine uptake was muscle specific, as it remained unchanged in TA (Figure 3I).

### 3.4 Prolonged stabilization of the ATF4 and downstream genes after exercise

**Figure 4:**
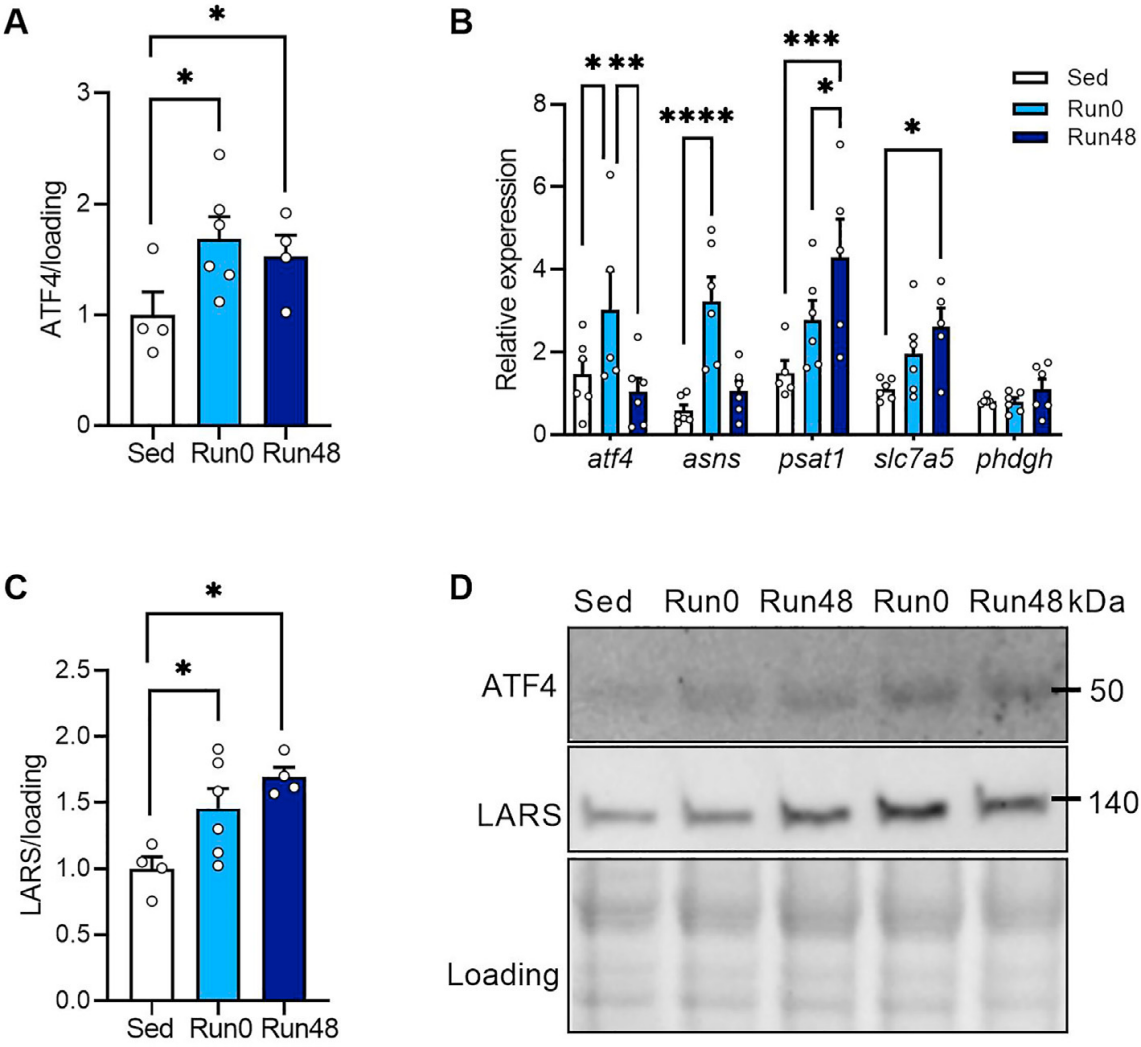
**ATF4 and downstream gene expression is increased after exercise.** (A) Quantification of ATF4. (B) qPCR of AA transporters in SOL. (C) Quantification of LARS. (D) representative immunoblots. Bars represent mean, circles represent individual values, error bars represent standard error of mean (SEM). All data is shown as fold change to Sed sal. Panel A–C, (Sed sal n = 4), (Run0 n = 6) (Run48 n = 4). One-Way ANOVA with Tukey's multiple comparisons test. ∗ p < 0.05; ∗∗ p < 0.01, ∗∗∗p < 0.001.

Previous work in other cell types has shown that the activating transcription factor 4 (ATF4) plays a key role in maintaining intracellular amino acid levels through regulating the expression of a variety of genes involved in amino acid uptake, sensing and metabolism [[47], [48], [49]]. We thus went on to investigate whether ATF4 was increased by exercise. ATF4 was barely detectable in non-exercised muscle, but was strongly increased in Run0 and Run48 in SOL (Figure 4A,D). This was not due to short term food withdrawal since we also observed higher ATF4 protein levels in Run0 and Run48 upon leucine feeding (Fig. S2A–D). Also, exercise did not affect ATF4 content in TA muscle (Fig. S2E–H). We found that several ATF4 target genes involved in amino acid metabolism were upregulated at Run0 (*asns*) or at Run48 (*psat1, slc7a5*) in SOL (Figure 4B), which combined with increased amino acid transporter expression, suggests increased ATF4-dependent transcriptional activity after exercise. We also measured the expression of leucyl tRNA synthetase (LARS), a putative leucine sensor in various cell types [16,21,50], which is controlled by ATF4 [51] and found a robust increase LARS protein levels in SOL at Run0 and Run48 (Figure 4C, D and Fig. S2B,D). Interestingly, in TA, which did not show enhanced mTORC1 signaling with exercise, LARS remained unaffected in any condition (Fig. S2F,H). Isoleucyl tRNA synthetase (IleRS), another aminoacyl-tRNA-synthetase that has no leucine-sensing properties remained stable at Run0 and Run48 (Fig. S2C, D, G and H), suggesting LARS specific regulation upon resistance exercise. These data show that ATF4 is activated up to 48 h after exercise cessation and suggest that ATF4 mediated activation of genes in amino acid uptake, metabolism and sensing might contribute to increased muscle leucine sensitivity.

### 3.5 mTORC1 suppression after exercise does not inhibit ATF4

**Figure 5:**
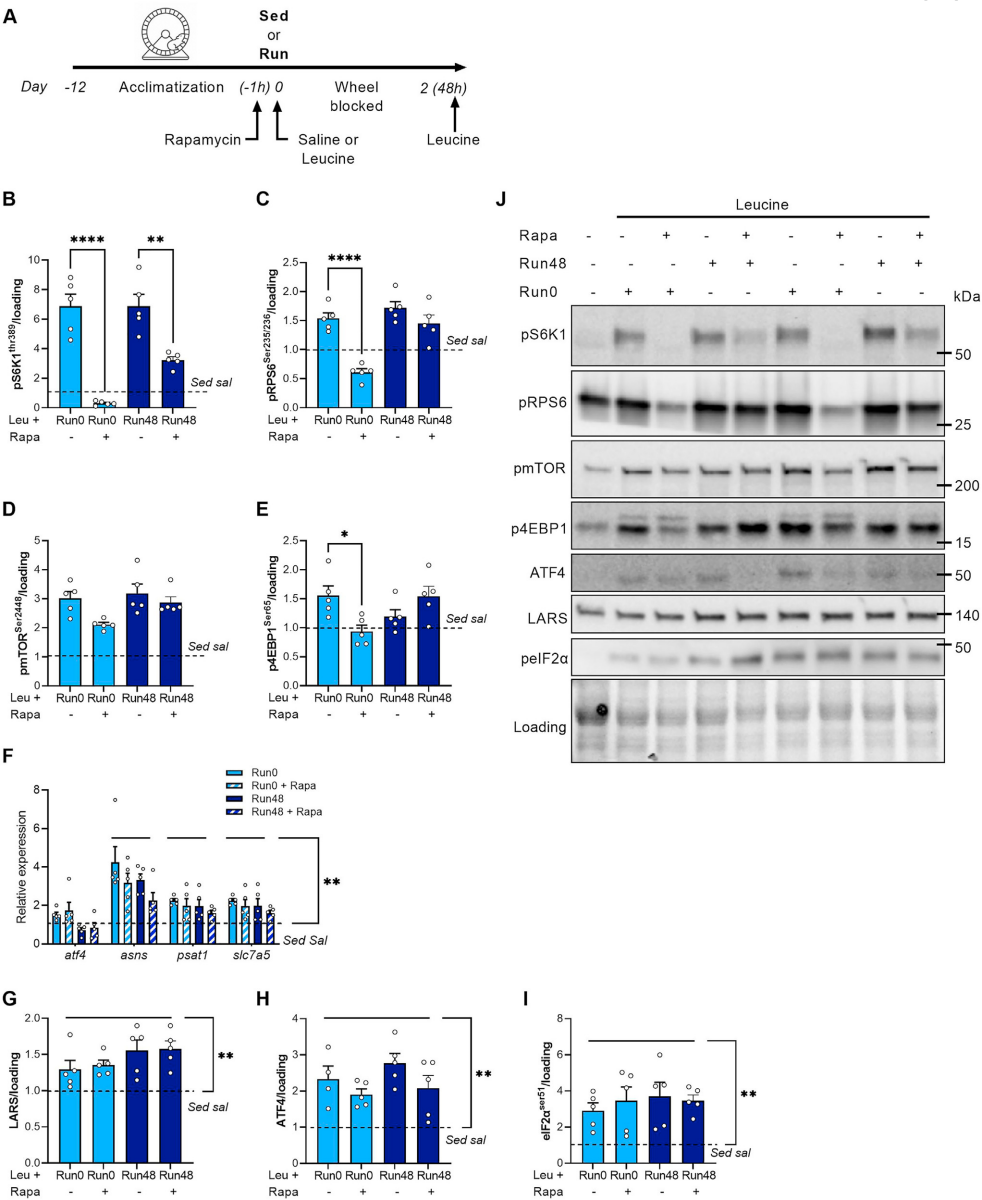
**Effect of rapamycin on mTORC1 and ATF4 after exercise and leucine.** (A) Experimental set-up. (B–E) Bar graphs showing downstream kinases of mTORC1 in SOL; (B) quantification of pS6K1^Thr389^, (C) Quantification of pRPS6^Ser235/236^, (D) quantification of mTOR^Ser2448^, (E) quantification of 4E-BP1^Ser65^. (F) qPCR of ATF4- dependent genes upon exercise and acute rapamycin administration. (G) Quantification of LARS. (H) Quantification of ATF4. (I) Quantification of eIF2α^Ser51^. (J) Representative immunoblots of downstream mTORC1 signaling and ATF4 pathway. Bars represent mean, circles represent individual values, all data is shown as fold change to Sed sal (dashed horizontal line). Panel B–E and G-I, (Sed sal n = 6), (Run0 n = 5), (Run0+rapa n = 5), (Run48 n = 5), (Run48+rapa n = 5). One-Way ANOVA with Tukey's multiple comparisons test. ∗ p < 0.05; ∗∗ p < 0.01, ∗∗∗p < 0.001. ∗∗∗∗p < 0.0001.

ATF4 is a transcription factor that is upregulated under conditions of stress where it acts downstream of eIF2α [52]. Recent studies however indicate that mTORC1 can activate ATF4 through mechanisms distinct from its canonical induction by the integrated stress response (ISR) [47]. Under those conditions, ATF4 acts as a critical downstream anabolic effector of mTORC1. Hence, to identify possible upstream regulation of ATF4, we first assessed whether the contraction-induced activation of mTORC1 is necessary to sustain long-term sensitivity towards leucine through ATF4 activation. To test this, we suppressed mTORC1 signaling by injecting the mTORC1 inhibitor rapamycin [18,53]. Preliminary experiments in Sed mice showed that a single injection (1.5 mg kg^−1^) sufficed to block mTORC1 activation for 48 h (Fig. S3). We subsequently injected rapamycin 2h after exercise in Run mice and assessed mTORC1/ATF4 expression at Run0 (60 min after rapamycin injection) and Run48 (Figure 5A). Results showed that leucine/Run mediated activation of mTORC1 signaling was completely blocked in Run0 (Figure 5B–E,J), but partially recovered in Run48 when rapamycin was administered (Figure 5B–E,J). Nonetheless, despite prolonged mTORC1 suppression, ATF4 and LARS expression remained elevated in Run0 and Run48 and were not affected by rapamycin at any point (Figure 5G–H,J), showing that ATF4 is not downstream of mTORC1 in this *in vivo* setting. This was further confirmed by the inability of rapamycin to suppress multiple ATF4-dependent target genes, both acutely and 48 h after exercise (Figure 5F). Importantly, increased ATF4 stability was *not* caused by mTORC1 activation during exercise, since blocking mTORC1 before exercise using an additional rapamycin injection (in addition to 2h after exercise) did not prevent the increase in ATF4 content (Fig S4a–d). Finally, since ATF4 and its downstream targets were not affected by rapamycin, we evaluated whether Run activated a prolonged stress response which could induce ATF4 expression via the activation of eIF2α, its canonical upstream regulator [52]. We found that eIF2α phosphorylation at Ser51 was increased at Run0 when compared to Sed and remained elevated at Run48 (Figure 5I,J). This suggests that the ISR rather than acute mTORC1 activation drives long-term ATF4 expression and LARS stabilization in skeletal muscle after exercise.

## 4 Discussion

Exercise and amino acids synergistically stimulate mTORC1 to maximize its activity [54]. Yet, this interaction has only been studied in a scenario where amino acids are provided shortly after the exercise stimulus and longer-term effects are poorly understood. In this study, we used a physiological exercise model in rodents that has been validated to induce robust mTORC1 activation [31] and muscle hypertrophy [41]. We subsequently fed a submaximal dose of leucine acutely and 48 h after exercise to study the long-term sensitizing effects of exercise towards mTORC1 signaling and MPS. Leucine feeding augmented downstream mTORC1 signaling acutely after exercise and, intriguingly, this potentiating effect remained intact at least 48 h after exercise cessation. Intramuscular free leucine levels correlated strongly to the increase in mTORC1 signaling and ^14^C leucine uptake remained elevated 48 h after exercise. Furthermore, exercise induced an increase in ATF4 protein content, which, together with increased LARS expression, suggests that muscular contractions switch on a coordinated program to enhance amino acid uptake as well as intramuscular sensing of key amino acids involved in mTORC1 activation and the stimulation of MPS. This enhanced sensitivity towards leucine remains elevated up to 48 h after exercise, signifying a much longer ‘anabolic window of opportunity’ than initially proposed [55,56].

In humans, exercise induces leucine oxidation [57,58], while simultaneously enhancing leucine uptake [29]. We show that when mice ran in a wheel against resistance for one night, baseline intramuscular leucine levels modestly increased (1.5-fold). Hence, as essential amino acids cannot be synthesized *de novo*, this suggests that leucine influx from the plasma and/or autophagic/proteasome-mediated protein degradation breakdown of muscle proteins outpaces leucine oxidation during and acutely after exercise. Interestingly, when we exogenously supplemented leucine, intramuscular leucine increased to a much larger extent (∼4-fold compared to Sed Sal), and this effect remained intact when leucine was supplemented 48 h after exercise. Our data support and further extends previous reports in humans [30] demonstrating that increased amino acid transporter mRNA and protein expression following a bout of resistance exercise may be an adaptive mechanism to increase amino acid import during postexercise recovery when combined with a subsequent anabolic stimulus. It is unclear whether these adaptations also affected baseline (Saline) mTORC1 activity. While intracellular free leucine and pS6K1 levels were similar to Sed conditions, we did find slightly higher pRPS6^Ser240/244^ at Run48.

We previously showed that resistance running does not increase mTORC1 signaling in dorsi flexors such as *m. tibialis anterior* (TA), which are recruited to a limited extent during resistance wheel running [31]. Consistent with those observations, we failed to observe a synergistic effect of leucine and exercise on mTORC1 activity. On the other hand, PLT muscle is recruited during resistance running in mice, as evidenced by increased mTORC1 activity at Run0 as well as its ability to undergo hypertrophy in response to 8-weeks of voluntary resistance running [41,42]. We however did not observe a synergistic effect of leucine and exercise in PLT at Run0 nor Run48. We can at this moment only speculate about the potential reasons for this discrepancy: First, we found that the activation of mTORC1 in response to leucine was very robust in PLT, potentially leading to maximal activation of mTORC1 already upon leucine supplementation. Second, while SOL contains more slow oxidative type I/IIa fibers, while PLT has a higher frequency of fast glycolytic type IIb/x fibers [41]. These fibers might differentially respond to leucine stimulation. In this respect, we already reported that oxidative fibers are particularly activated during exercise, likely due to a lower recruitment threshold [31]. In that study, mTORC1 activation upon exercise in PLT was also lower when compared to SOL. Thus, loading or recruitment of plantaris might be less pronounced when compared to soleus loading. Potentially, the load/recruitment threshold for ‘synergistic’ activation at Run48 was not reached in PLT.

In our study, exercise increased exogenous amino acid influx long-term. Hence, we wondered whether the capacity to sense the enhanced intracellular levels would be altered by exercise as well. We observed a strong increase in ATF4, which induces enhanced transcription of genes involved in amino acid transport and purine synthesis to relieve cellular stress [59], acutely and 48 h after exercise. This coincided with a similar induction of LARS, a putative leucine sensor under control of ATF4 [51]. Recent reports suggest that LARS-leucine binding is fully saturated at physiological intracellular leucine concentrations [21], questioning its role in leucine sensing *in vivo* [24]. Nevertheless, although we did not measure the specific binding of leucine to LARS, our data suggest that leucine sensing towards mTORC1 is elevated due to a robust increase in protein stability of LARS, mediated by exercise. Future studies should further explore how exercise affects leucine binding to LARS and whether LARS protein stability plays a causal role in anabolic resistance with aging [60].

Activation of mTORC1 governs a coordinated anabolic program in response to growth signals. Paradoxically, recent *in vitro* data showed that upon insulin stimulation, mTORC1 itself can activate ATF4 distinct from its induction by the ISR [47,49]. This is intriguing as ATF4 had been previously linked to muscle atrophy when induced long-term [61,62]. The mTORC1-ATF4 axis stimulates only a subset of ATF4-dependent target genes involved in amino acid uptake and, interestingly, tRNA charging [47]. We did not observe an increase in ATF4 upon leucine feeding in sedentary conditions (Fig. S2A). Moreover, we used the rapamycin injections to understand whether mTORC1 acts upstream of ATF4 in our model. While mTORC1 has rapamycin-insensitive roles during hypertrophy [63], previous data showed that the mTORC1-dependent increase in ATF4 is to a large extent rapamycin-sensitive [18,19]. As visible from our experiments and shown before by others [18] rapamycin potently blocked the acute activation of mTORC1 following exercise. Blocking mTOR however did not affect the increase in ATF4 nor LARS content, strongly suggesting that mTORC1 is not the dominant upstream regulator of ATF4 in our experimental setting.

In contrast, we found a robust increase in eIF2α Ser51 phosphorylation after exercise, indicating that one bout of high-intensity exercise increases ATF4 via IRS rather than directly via mTORC1. Earlier work by Damas and coworkers in humans suggested that much of the protein synthesis response early after acute exercise could be a consequence of muscle damage [64]. Furthermore, one week of unweighted wheel running in mice was sufficient to cause ∼60% of the fibers to be centrally nucleated and 20% to express embryonic myosin heavy chain (eMHC) [65]. Interestingly, we did not find any eMHC expression in both SOL and PLT in our study set-up (data not shown). We hypothesize this is because 1/we acclimatized the mice with sufficient recovery between running nights (2–3 days). Additionally, 2/one night of running might be insufficient to cause micro damage to the muscle fibers which leads to eMHC expression. Although we cannot completely rule out that muscle damage *per se* leads to the activation of the integrated stress response ISR in SOL, many other exercise-induced signals such as calcium depletion, energetic or nutrient stress, and hypoxia could upregulate pEIF2α and ATF4 in SOL independent of damage [66,67]. Altogether, our data show that the IRS is a major driver of prolonged mTORC1 activation after exercise in combination with leucine feeding.

The question remains why ATF4 and mTORC1 were simultaneously increased in the hours and days after exercise, especially when amino acids were provided exogenously. It is tempting to speculate that ATF4 expression and the subsequent increase in amino acid influx after exercise may be a coordinated program to fully amplify mTORC1 activation and muscle anabolic adaption upon amino acid feeding. As a consequence, an impaired contraction-induced stress response could blunt mTORC1 activation. In this respect, although there is a robust activation of mTORC1 acutely after one bout of muscle contractions [17,18], training the muscle via multiple exercise sessions diminishes mTORC1 activation over time [68]. Moreover, 5 weeks high intensity exercise relieved ER stress markers in rats [69], while the pathway is activated acutely after ultra-endurance exercise [70]. As such, future *in vivo* studies should explore whether a relief of cellular stress (ISR) is causally related to diminished mTORC1 activation with chronic exercise training and hence, muscle remodeling.

Finally, we previously observed that in muscle endothelial cells, endothelial ATF4 is required for the increased vascular density upon exercise training [48]. Together with our current observations, we speculate that ATF4 might be a crucial anabolic effector that allows cell growth (whether muscle growth or endothelial growth for proliferation) following exercise.

In conclusion, we show that exercise enhances muscle sensitivity for leucine acutely and up to 48 h after exercise, which drives mTORC1 activation and MPS. This coincided with a long-term increase in leucine uptake and enhanced expression of ATF4 and LARS. Finally, an acute pharmacological block of mTORC1 did not lower ATF4 expression indicating that muscle contractions do not drive mTORC1-dependent ATF4 transcription. The present findings show that exercise and amino acids synergistically activate mTORC1 and provide further evidence that the anabolic window of opportunity is much more extended than initially thought.

## Author contributions

GDH: Conceptualization, data curation, experimentation, writing original draft, figure design, review and editing. EM: Conceptualization, figure design, manuscript review and editing. KDB: Conceptualization, data curation, funding acquisition, supervision, writing original draft, figure design, review and editing.

## Data Availability

Data will be made available on request.
