# Supplementary material for: Resistance exercise enhances long-term mTORC1 sensitivity to leucine
Source: Mol Metab. 2022 Oct 14;66:101615. doi: 10.1016/j.molmet.2022.101615 (PMC9626937; doi:10.1016/j.molmet.2022.101615)
Supplement: Multimedia component 1 [file mmc1.docx]

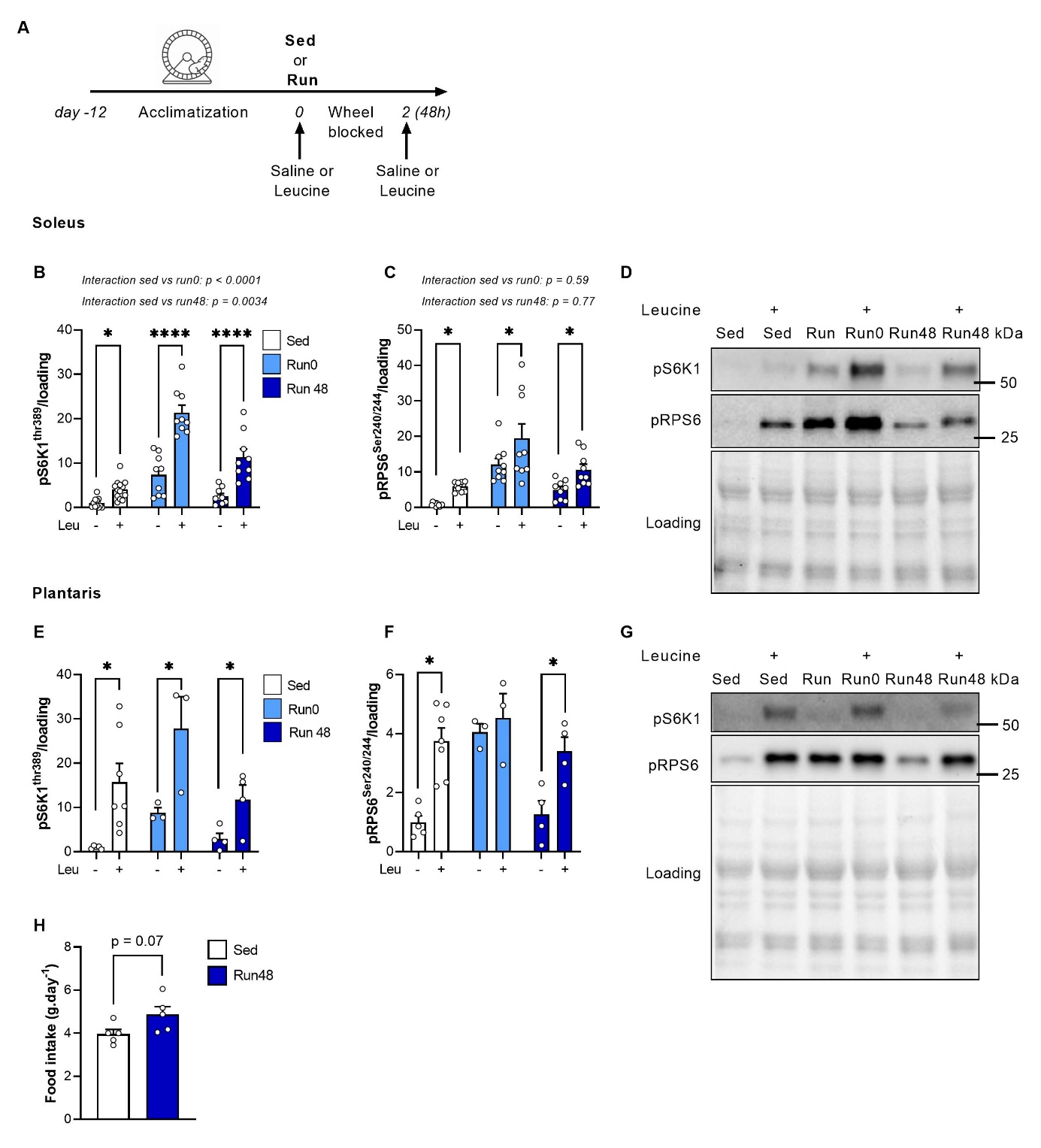


**Figure S1**. **Long-term activation of mTORC1 with exercise and leucine in SOL, not PLT.** (A) experimental protocol. Quantification (B-C) and representative immunoblots (D) of pS6K1^Thr389^ (B) and pRPS6^Ser240/244^ (C) in SOL. Quantification (E-F) and representative immunoblots (G) of pS6K1^Thr389^ (E) and pRPS6^Ser240/244^ (D) in PLT. Food intake in sedentary mice and during the two days after acute running (H). Bars represent mean, circles represent individual values, error bars represent standard error of mean (SEM). All data is shown as fold change to Sed sal (dashed horizontal line). Panel B-C, (Sed sal n=8), (Sed leu n=10) (Run0 w/o leucine n=9) (Run0 w leucine n=9) (Run48 w/o leucine n=9) (Run48 w leucine n=8). Panel E-F, (Sed sal n=5), (Sed leu n=7) (Run0 w/o leucine n=3) (Run0 w leucine n=3) (Run48 w/o leucine n=4) (Run48 w leucine n=4). Panel H, (Sed n=5), (Run48 n=10). Two-Way ANOVA with Tukey's multiple comparisons test (panel B,C, E and F). Student’s T-Test (panel H). ^∗^ p < 0.05; ^∗∗^ p < 0.01, ^***^ p < 0.001, ^****^ p < 0.0001.


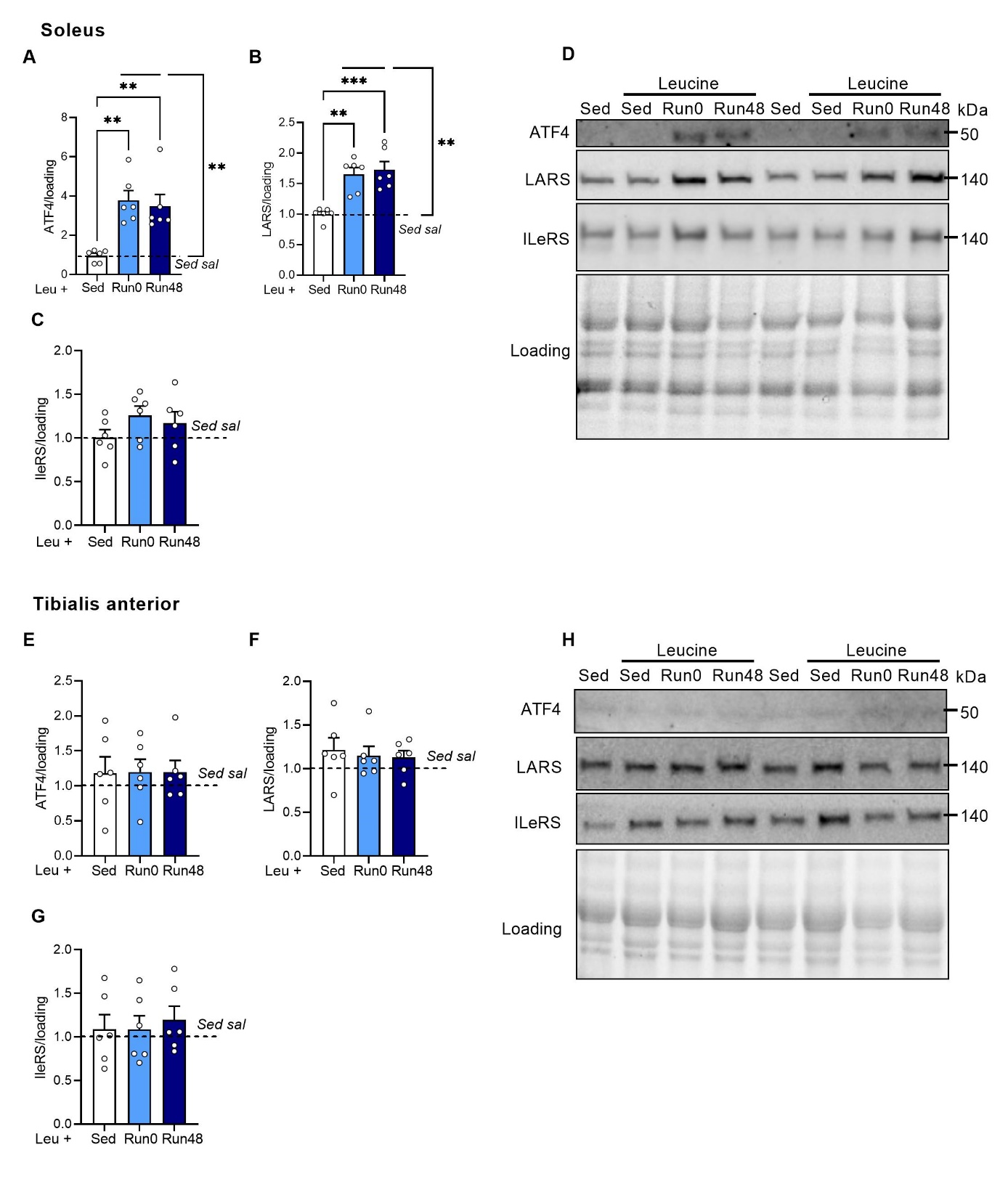


**Figure S2**. **ATF4 and downstream gene expression after combination of exercise and leucine supplementation in SOL and TA.** (A-D) Quantification (A-C) and representative immunoblots (D) of ATF4 (A), LARS (B), and IleRS (C) in SOL. Quantification (E-G) and representative immunoblots (H) of ATF4 (E), LARS (F), and IleRS (G) in TA. Bars represent mean, circles represent individual values, error bars represent standard error of mean (SEM). All data is shown as fold change to Sed sal (dashed horizontal line). Panel A-C and E-G, (Sed sal n=8), (Sed leu n=10) (Run0 n=6) (Run48 n=6). One-Way ANOVA with Tukey's multiple comparisons test (panel A-C, E and G). ^∗^ p < 0.05; ^∗∗^ p < 0.01, ^***^ p < 0.001.


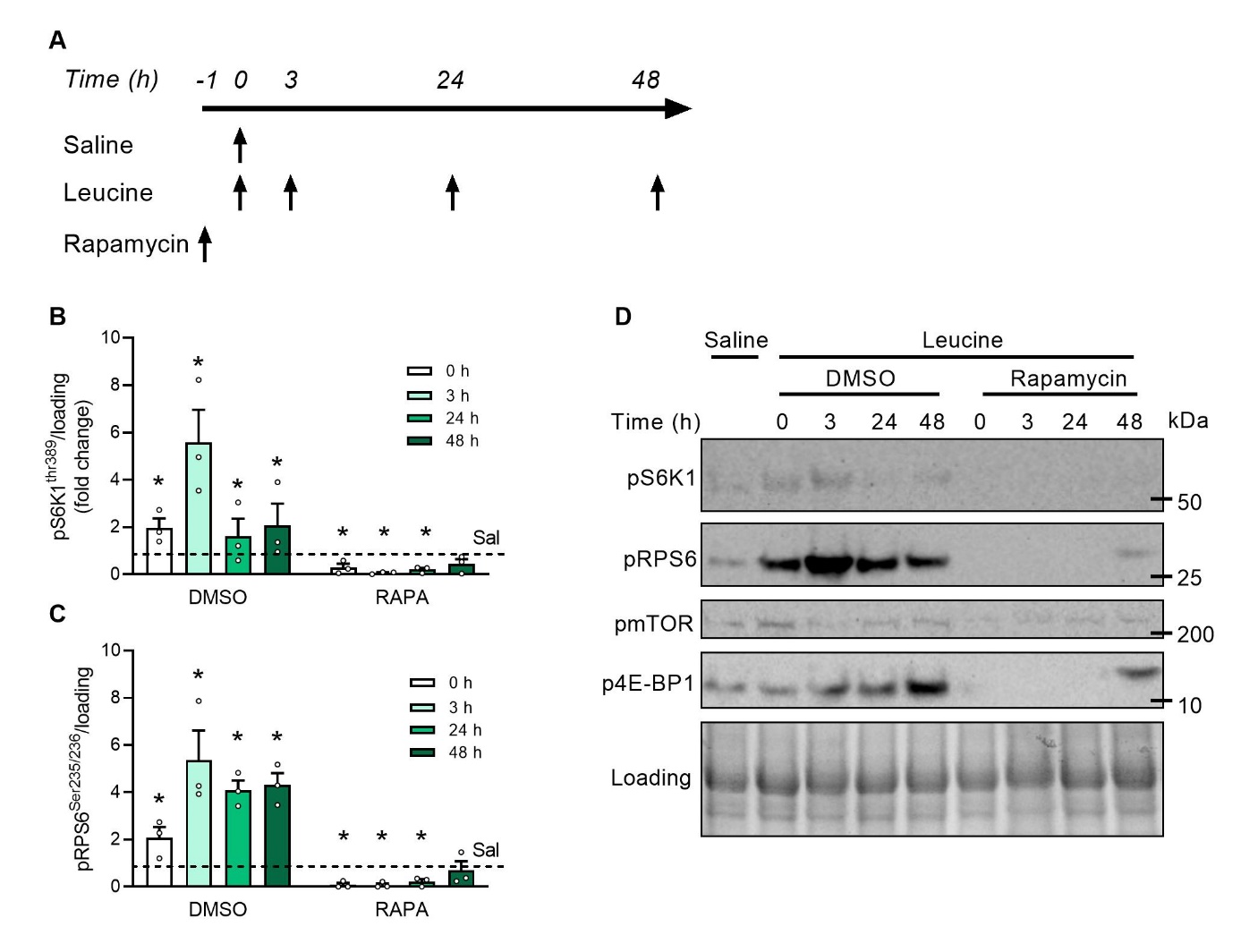


**Figure S3. Post-exercise rapamycin treatment leads to long-term mTORC1 inhibition** (A) Experimental set-up. (B-C) Bar graphs showing downstream kinases of mTORC1 in SOL; (B) quantification of pS6K1^Thr389^, (C) quantification of pRPS6^Ser235/236^. (D) Representative immunoblots of downstream mTORC1 signaling. Bars represent mean, circles represent individual values, error bars represent standard error of mean (SEM). All data is shown as fold change to Sed sal. Panel B-C, (Sed sal n=5), (time0 n=3), (time3 n=3), (time24 n=3), (time48 n=3). One-Way ANOVA with Tukey's multiple comparisons test. ^∗^ p < 0.05 vs Sed sal.


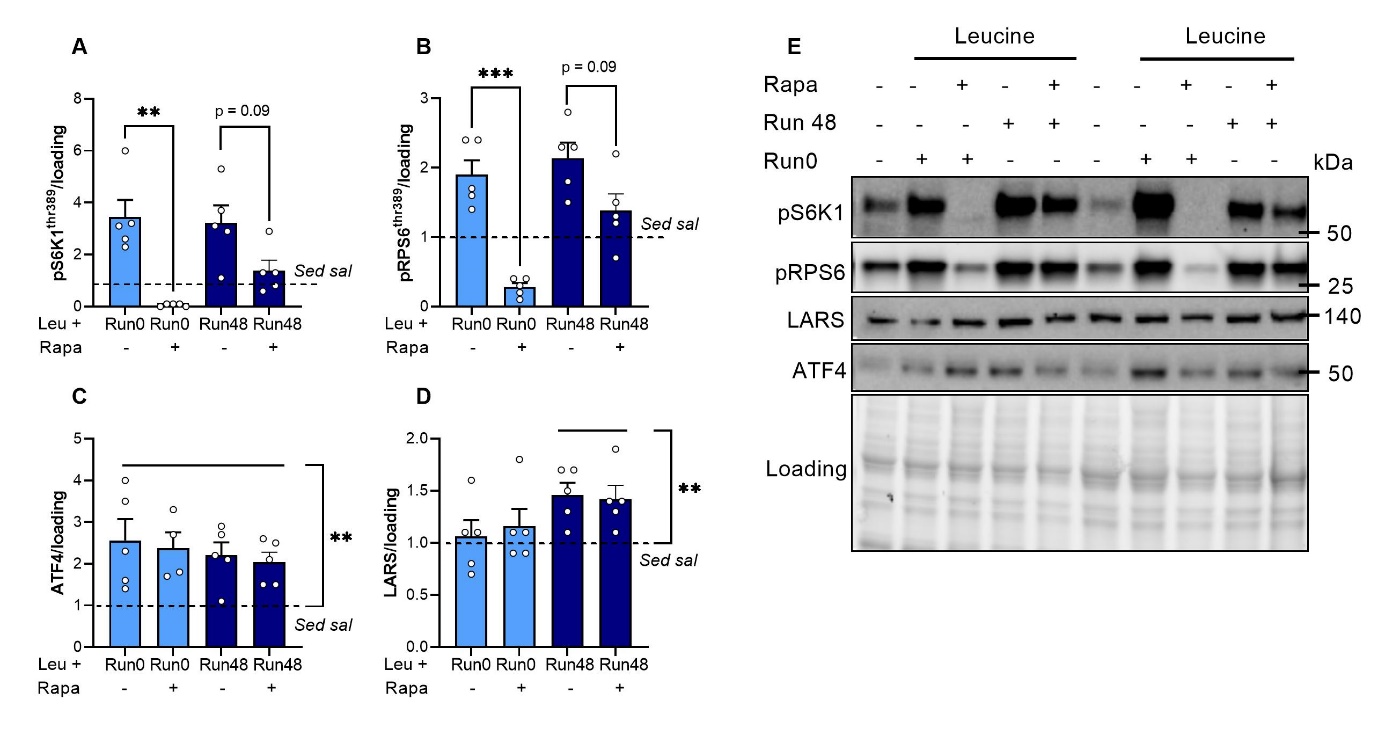


**Figure S4**. **Effect of double dose rapamycin on mTORC1 and ATF4 after exercise and leucine.** (A) quantification of pS6K1^Thr389^, (B) quantification of pRPS6^Ser235/236^, (C) quantification of ATF4, (D) quantification of LARS, (E) representative immunoblots. Bars represent mean, circles represent individual values, data is shown as fold change to Sed sal (dashed horizontal line). Panel A-D (n=5 all groups). One-Way ANOVA with Tukey's multiple comparisons test. ^∗^ p < 0.05; ^∗∗^ p < 0.01, ^***^ p < 0.001. ^****^ p < 0.0001.
